# Radcliffe Infirmary, Oxford

**Published:** 1894-12-01

**Authors:** B. W. Gowring, A. B. Boyd

**Affiliations:** House Surgeon; House Physician


### Editor's Letter-Box.

[Our correspondents are reminded that proxility is a great bar to publication, and that brevity of style and conciseness of statement greatly facilitate early insertion.]

### RADCLIFFE INFIRMARY, OXFORD.

Sir—We have read with a considerable amount of interest the article in The Hospital of this week on the Radcliffe Infirmary by your Special Commissioner, and especially that part of it dealing with the administration. Undoubtedly an investigation into this would be beneficial to the institution, even if it only dealt with the question, why it is that the last five or six residents have left before, and in some cases a considerable time before, the expiration of their term of office.
—Yours faithfully,

B. W. Gowring, House Surgeon.
A. B. Boyd, House Physician.

